# The myriad roles of Miro in the nervous system: axonal transport of mitochondria and beyond

**DOI:** 10.3389/fncel.2014.00330

**Published:** 2014-10-28

**Authors:** Kyu-Sun Lee, Bingwei Lu

**Affiliations:** Department of Pathology, Stanford University School of MedicineStanford, CA, USA

**Keywords:** mitochondrial rho GTPase, mitochondrial motility, mitochondrial morphogenesis, calcium homeostasis, mitochondria-ER communication, microtubule dynamics, apoptosis

## Abstract

Mitochondrial rho GTPase (Miro) is a mitochondrial outer membrane protein containing two GTPase domains and two helix-loop-helix Ca^2+^-binding domains called EF hands. Pioneering genetic studies in *Drosophila* first revealed a key function of Miro in regulating the axonal transport of mitochondria, during which Miro forms a multi-protein transport complex with Milton and Kinesin heavy chain (KHC) to link trafficking mitochondria with the microtubule (MT) cytoskeleton. Recent studies showed that through binding to the EF hands of Miro and causing conformational changes of Miro and alteration of protein-protein interactions within the transport complex, Ca^2+^ can alter the engagement of mitochondria with the MT/kinesin network, offering one mechanism to match mitochondrial distribution with neuronal activity. Despite the importance of the Miro/Milton/Kinesin complex in regulating mitochondrial transport in metazoans, not all components of the transport complex are conserved in lower organisms, and transport-independent functions of Miro are emerging. Here we review the diverse functions of the evolutionarily conserved Miro proteins that are relevant to the development, maintenance, and functioning of the nervous system and discuss the potential contribution of Miro dysfunction to the pathogenesis of diseases of the nervous system.

## Introduction

Mitochondrial rho GTPase (Miro) was initially identified by searching the public DNA and protein databases for novel members of the Rho GTPases family. These proteins have tandem GTP-binding domains separated by a linker region containing putative calcium-binding EF hand motifs (Fransson et al., 2003). Genes encoding Miro-like proteins have been found in all eukaryotes, from yeast* and Arabidopsis*, to *Drosophila* and mammals, indicating high degree of evolutionary conservation.

Genetic studies in *Drosophila* have revealed that Miro functions in a Miro/Milton/Khc complex to transport neuronal mitochondria (Stowers et al., 2002; Guo et al., 2005; Pilling et al., 2006). The homologous Miro/TRAK/KIF5 complex serves similar roles in mammalian neurons (Hirokawa et al., 1991; Brickley et al., 2005; Fransson et al., 2006). Recent studies showed that the EF hands of Miro can sense Ca^2+^ influxes evoked by synaptic activation, causing conformation changes in Miro and altered protein-protein interactions within the transport complex, leading to mitochondrial immobilization at active synapses (Saotome et al., 2008; MacAskill et al., 2009b; Wang and Schwarz, 2009). This offers one elegant mechanism to match mitochondrial distribution with intracellular Ca^2+^ levels.

Despite the importance of the Miro/Milton/Khc complex and its mammalian counterpart in trafficking neuronal mitochondria in metazoans, disruption of the single Miro-encoding gene in *Dictyostelium discoideum* did not significantly affect mitochondrial size and distribution, although decreased mitochondrial mass and cellular adenosine triphosphate (ATP) content were observed (Vlahou et al., 2011). It thus appears that in addition to regulating mitochondria motility, Miro may perform other important functions in mitochondria physiology. Given the importance of mitochondria to neuronal function and the association of mitochondrial dysfunction to a large number of neurodegenerative and neuropsychiatric disorders such as Parkinson’s disease (PD), Alzheimer’s disease (AD), and Schizophrenia (Wallace, 2005; Beal, 2007; Mattson et al., 2008), a complete understanding of the roles of Miro proteins is highly relevant to human health and disease. Here we review the multitude functions of Miro as reveled by studies in diverse organisms and discuss their implications towards our understanding of the roles of mammalian Miro proteins in the development, functioning, and maintenance of the nervous system.

## The Miro/Milton/Kinesin complex in axonal transport of mitochondria

The distribution and morphology of mitochondria need to adequately adapt to environmental signals and changing metabolic states of the cell. Dynamic mitochondria are particularly important to the brain, which, despite making up only 2% of the human body mass, consumes ~20% of the body’s resting energy production, making brain one of the most energy-demanding tissues (Tomasi et al., 2013). In neurons, mitochondria not only supply energy but also play critical roles in synapses to buffer Ca^2+^ influxes elicited by neurotransmission and to promote synaptic differentiation and plasticity (Saxton and Hollenbeck, 2012; Sheng and Cai, 2012). Given the dynamic nature of neuronal activity patterns and of synaptic plasticity, precise regulatory mechanisms are required to distribute mitochondria appropriately to subcellular sites where these organelles are needed. Our understanding of the molecular and cellular mechanisms regulating the precise distribution of mitochondria in neurons remains rather limited.

**Figure 1:**
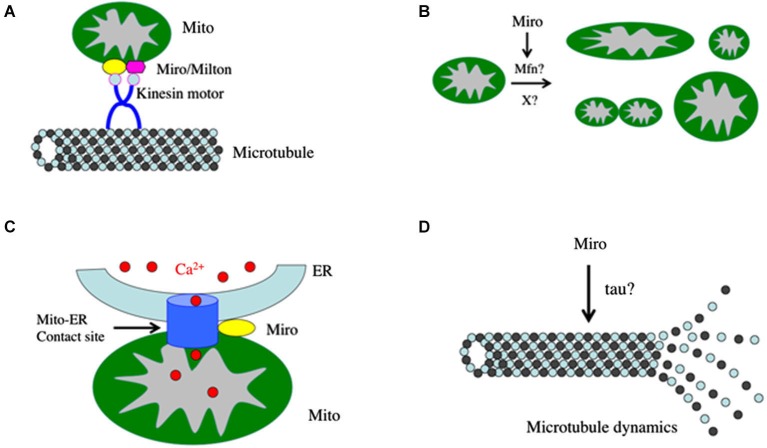
**Diagram depicting possible roles of Miro in the nervous system. (A)** Miro forms a complex with Milton and kinesin heavy chain to mediate mitochondrial transport along the microtubule cytoskeleton. **(B)** Miro influences mitochondrial morphology, presumably through its interaction with Mitofusins or some unknown factors. **(C)** Miro regulates the integrity or function of the mitochondria-ER contact sites, which mediate calcium transfer from the ER to mitochondria through calcium transporters. **(D)** Miro regulates microtubule dynamics through an unknown mechanism. This aspect of Miro function may affect the cell cycle and division of neural stem cells.

In a genetic screen for mutations that affect synaptic structure and function in *Drosophila* (Guo et al., 2005), Zinsmaier et al. identified ethyl methane sulfonate (EMS)-induced mutations in *Drosophila* Miro (dMiro). The authors observed that mitochondria in *dmiro* mutant muscles and neurons are abnormally distributed. They accumulate in neuronal soma instead of being transported into axons and dendrites. As a result, mutant neuromuscular junctions (NMJs) lack presynaptic mitochondria, and neurotransmitter release and acute Ca^2+^ buffering is impaired during prolonged high frequency stimulation but not under basal conditions. Conversely, gain of *dMiro* function causes an abnormal accumulation of mitochondria in distal synaptic boutons of NMJs, supporting that dMiro promotes anterograde transport of mitochondria and their proper distribution within nerve terminals. A similar phenotype was observed in the *Drosophila milton* mutant, in which mitochondria are missing from synaptic terminals and axons, but abundant in cell bodies (Stowers et al., 2002). At the biochemical level, Milton protein was found to associate with mitochondria and coimmunoprecipate with Kinesin heavy chain (KHC), suggesting that Milton is a mitochondria-associated protein required for kinesin-mediated transport of mitochondria to nerve terminals (Stowers et al., 2002). The similar mitochondrial localization of Milton and Miro proteins and their similar mutant phenotypes suggest that Milton and Miro may act in a common protein complex. Glater et al showed that the microtubule (MT)-dependent transport of mitochondria depends on the ability of milton to act as an adaptor protein that can recruit KHC to mitochondria (Glater et al., 2006). Moreover, the authors demonstrated a direct interaction between Milton and Miro, and this interaction can influence the recruitment of Milton to mitochondria, suggesting that there likely exists a Miro/Milton/KHC complex that recruit KHC to mitochondria for MT-dependent anterograde transport of mitochondria (Figure 1). Russo et al used live-imaging to monitor the movements of GFP-tagged mitochondria in the axons of *Drosophila* larval motor neurons and found that loss of Miro function reduced the effectiveness of both anterograde and retrograde mitochondrial transport by selectively impairing kinesin- or dynein-mediated movements, depending on the direction of net transport (Russo et al., 2009). Interestingly, overexpression of dMiro also impaired the effectiveness of mitochondrial transport, although the molecular basis of the overexpression effect is unclear. The authors proposed that dMiro might promote effective antero- and retrograde mitochondrial transport by extending the processivity of kinesin and dynein motors according to the organelle’s programmed direction of transport (Russo et al., 2009). A recent study showed that the mammalian Milton homologs, TRAK1 and TRAK2, are required for axonal and dendritic mitochondrial motility and that they utilize different transport machineries to move mitochondria into axons and dendrites. TRAK1 binds to both kinesin-1 and dynein/dynactin and is prominently localized in axons, whereas TRAK2 predominantly interacts with dynein/dynactin and is more abundantly present in dendrites (van Spronsen et al., 2013). The interaction of TRAK1 with both the kinesin and dynein motors may confer Miro and the transport machinery the ability to regulate both antero- and retrograde mitochondrial transport in axons. It remains to be seen whether Milton interacts with both the kinesin and dynein motors in *Drosophila*.

Studies in mammalian neurons have largely supported the conservation of the Miro/Milton/KHC transport machinery in directing mitochondrial trafficking. The closest Milton-related proteins in humans are the coiled-coil domain containing proteins TRAK1 and TRAK2, which have been reported to interact with kinesin-1 family members (Brickley et al., 2005; Smith et al., 2006). TRAK1 and TRAK2 co-localize with mitochondria and interact with hMiro1 and hMiro2 in cultured cells. Their over-expression led to the formation of aggregated and thread-like mitochondria, similar to the effect of hyperactive hMiro1. In biochemical assays, hMiro1 was shown to associate with TRAK1 and TRAK2. This interaction was independent of the GTPase state or the capability of the EF-hands of Miro to bind calcium, but may depend on the first GTPase domain of Miro. The physiological function of Miro, TRAK, and kinesin proteins in regulating neuronal mitochondrial transport in mammals was demonstrated in rat hippocampal neuronal culture (MacAskill et al., 2009b; Wang and Schwarz, 2009; Brickley and Stephenson, 2011). hMiro1 and TRAK2 were shown to form a protein complex in mammalian brain tissue extracts and co-localize to neuronal mitochondria. Increasing hMiro1 expression facilitated the recruitment of TRAK2 to mitochondria and promoted the anterograde transport of mitochondria, whereas uncoupling hMiro1 from the kinesin motor (KIF5) proteins by deleting the kinesin-binding domain in TRAK2 inhibited the anterograde transport of mitochondria into distal processes. Interestingly, altering Miro1 function by mutating its first GTPase domain impaired Miro’s ability to recruit TRAK2 to mitochondria and altered mitochondrial distribution and shape along neuronal processes (MacAskill et al., 2009a). Thus, mitochondrial motility in mammalian neurons is controlled by a mechanism that is dependent on the GTPase activity of Miro for the recruitment of TRAK to mitochondria and on the connection to the MT network through kinesin motors. In addition to their interactions with KHC and Miro, both TRAK1 and TRAK2 associate with O-GlcNAc transferase (OGT), an enzyme catalyzing post-translational O-glycosylation (Brickley et al., 2011). Recent proteomic studies suggest that TRAKs are modified by O-GlcNAc (Trinidad et al., 2012). Extracellular glucose was shown to activate OGT, which arrests mitochondrial motility through TRAK GlcNAcylation. TRAK/Milton GlcNAcylation by OGT thus links neuron mitochondrial motility to nutrient availability (Pekkurnaz et al., 2014).

In addition to bioenergetics, mitochondria play other physiological roles essential for neuronal function and maintenance, such as buffering of intracellular Ca^2+^ levels. The conservation of the Ca^2+^ binding EF hands in Miro proteins from yeast to humans raised the possibility that Miro is involved in Ca^2+^ signaling or homeostasis. Three independent studies identified Miro as a key Ca^2+^ sensor in Ca^2+^-dependent arrest of mitochondrial trafficking in both axons and dendrites in cultured neurons (Saotome et al., 2008; MacAskill et al., 2009b; Wang and Schwarz, 2009). This regulatory function of Miro on mitochondrial trafficking is dependent on the EF-hand domains, which bind Ca^2+^ upon neuronal activity-induced Ca^2+^ influx and allow mitochondria to accumulate at sites in need of energy or Ca^2+^ buffering (e.g., the active synapses). Despite the agreement on the essential role of Miro in sensing Ca^2+^ and confer Ca^2+^-dependent arrest of mitochondrial motility, there is less consensus on the detailed molecular mechanisms by which Ca^2+^ regulates mitochondrial motility. Several models have been proposed. In one model, it is proposed that increased Ca^2+^ causes the KIF5 to dissociate from microtubules and to interact with Miro on mitochondria, dissociating the motors from microtubules (Wang and Schwarz, 2009). In another model, increased Ca^2+^ levels inhibit the interaction between Miro and KIF5, thus directly uncoupling Miro and mitochondria from the MT motor system (MacAskill et al., 2009b). Another study proposed that Miro plays a key role in regulating intramitochondrial Ca^2+^ levels in the matrix, and that intramitochondrial Ca^2+^, rather than cytoplasmic Ca^2+^, plays a key role in determining mitochondrial transport (Chang et al., 2011). A recent study further implicated the mitochondrial tethering protein syntaphilin (SNPH) in Miro and Ca^2+^-dependent arrest of mitochondrial trafficking in neuronal axons. In a so-called “Engine-Switch and Brake” model, it is proposed that increased Ca^2+^ level dissociates KIF5 from Miro, allowing KIF5 to interact with MT-bound SNPH, and resulting in inhibition of the ATPase activity of KIF5 by SNPH. Syntaphilin therefore acts both as an engine-off switch by sensing Miro/Ca^2+^-dependent mitochondrial arrest and as a brake by tethering stationary mitochondria to the MT track (Chen and Sheng, 2013). It is likely that these diverse models are not mutually exclusive. Further studies are clearly needed to figure out the exact molecular mechanisms underlying the regulatory roles of Ca^2+^ and Miro on mitochondrial transport in axons and dendrites, and to identify addition Ca^2+^-independent mechanisms that regulate Miro function in mitochondrial transport.

## Regulation of mitochondrial morphology by Miro

In addition to participating in mitochondrial transport, Miro also regulates mitochondrial morphology in all organisms examined, from yeast and flies, to plants and mammals (Fransson et al., 2003; Frederick et al., 2004; Russo et al., 2009), although the underlying mechanism is still largely unknown. In the first functional study of Miro function in mammalian cells, overexpression of a constitutively active form of hMiro1 resulted in collapsing of the mitochondrial network and the aggregation of mitochondria into perinuclear assemblies. Overexpression of a dominant-negative form of hMiro1 had similar effect, albeit to a lesser extent (Fransson et al., 2003). These results indicate that the appropriate level of Miro activity is important for mitochondrial morphology. In plants, the *Arabidopsis thaliana* genome encodes two Miro-related proteins, Miro1 and Miro2, which were shown to be ubiquitously expressed in plant tissues and to localize to the outer mitochondrial membrane. Mutations in the Miro1 gene were found to result in abnormally enlarged or tube-like mitochondrial morphology in the growing pollen tube, supporting that the function of Miro in controlling mitochondrial morphology is conserved in plants (Yamaoka and Leaver, 2008). The yeast homolog of Miro, Gem1p, is a tail-anchored outer mitochondrial membrane protein. Cells lacking Gem1p exhibit collapsed, globular, or grape-like mitochondria. Structure-function studies indicated that both the GTPase domains and EF-hand motifs are required for Gem1p function in regulating mitochondrial morphology (Frederick et al., 2004). Although these results support a conserved role of Miro in regulating mitochondrial morphology, yeast Gem1p does not appear to be a key component of the conserved fission and fusion machineries (Frederick et al., 2004). The function of Miro in regulating mitochondrial morphology is also observed in *Drosophila*. Overexpression of wild type Miro resulted in elongated mitochondria in *Drosophila* larval motor neurons (Russo et al., 2009) and aggregated mitochondria in dopaminergic neurons (Liu et al., 2012), whereas inhibition of Miro function altered mitochondrial motility and distribution but had no obvious effects on mitochondria morphology. However, the loss of function effect of Miro on mitochondrial morphology could be cell type- and context-dependent *in vivo*, and more thorough analysis is required to address the physiological role of Miro in mitochondrial morphogenesis.

The molecular mechanism by which altered Miro function affects mitochondrial morphology is not well understood. It is generally thought that the probability of a single mitochondrion fusing is determined primarily by its motility, i.e., a motile mitochondrion has better chances of finding fusion partners. This might explain the excessive mitochondrial aggregation or fusion phenotypes induced by Miro overexpression. Conversely, one could envision that mitochondria size and motility may also be mechanistically linked, e.g., smaller mitochondria might be able to move faster than bigger ones. However, experimental support for this latter assumption has been lacking. Genetic manipulations that alter mitochondrial size had no obvious effect on mitochondrial transport (Liu et al., 2012). A study in mammalian systems identified physical interaction between the pro-fusion proteins mitofusins (Mfn1 and Mfn2) and Miro (Miro1/Miro2) or Milton (TRAK1/TRAK2) proteins (Misko et al., 2010). It remains to be determined whether the Miro/Mfn interaction may contribute to the effect of Miro on mitochondrial morphology. Genetic studies indicated that the pleotropic effects of yeast Miro (Gem1p) on mitochondrial morphology are not mediated by the canonical fission and fusion pathways (Frederick et al., 2004), suggesting that Miro may act in a novel mitochondrial morphogenesis pathway (Figure 1). Future genetic studies in model organisms promise to dissect a potentially novel mitochondrial morphogenesis pathway mediated by Miro.

## Regulation of mitochondrial Ca^2+^ homeostasis by Miro at ER-Mito contact sites

Recent studies in yeast have revealed an interesting subcellular localization of Miro to the mitochondrial-endoplasmic reticulum (ER) contact sites (Kornmann et al., 2011). Yeast mitochondria are connected to the ER through the ER-mitochondria encounter structure (ERMES) tethering complex. ERMES is known for its involvement in phospholipid exchange between the two organelles, but has also been implicated in coordinating mitochondrial protein import, mitochondrial DNA replication, and mitochondrial dynamics, suggesting profound impacts of these structures on organellar physiology. Yeast Gem1 was found to be as an integral component of ERMES and regulate the number and size of the ERMES complexes. Importantly, mammalian Miro-1 was also found to localize to sites of ER-mitochondrial contact, suggesting evolutionary conservation and functional importance of ERMES in mediating Miro function (Kornmann et al., 2011). At least in yeast, the ER-mitochondria contact sites mark mitochondrial division sites. A recent study showed that the ERMES and Gem1 are spatially and functionally linked to ER-associated mitochondrial division, whereby Gem1 acts as a negative regulator of ER-mitochondria contact, an activity required for the spatial resolution and distribution of newly generated mitochondrial units following division. These results suggest a role for ERMES and Gem1 in regulating the distribution of mitochondria and mitochondrial DNA during organelle division and biogenesis in yeast (Murley et al., 2013). Since Gem1p is the only ERMES constituent that can be unambiguously identified in metazoans, it remains to be determined whether the multitude of ERMES functions uncovered in yeast are relevant to Miro function in higher organisms.

One possible function of Miro proteins localized at the mitochondria-ER contact sites is to regulate mitochondria-ER Ca^2+^ signaling (Figure 1). At those contact sites, Ca^2+^ is released from the ER to mitochondria, a process that is important for mitochondrial function. In addition to providing a buffering system for cytoplasmic Ca^2+^, intramitochondrial Ca^2+^ is needed for ATP production by activating the TCA cycle enzymes and enhancing the activities of the electron transport chain complexes and the ATP synthase complex (Wan et al., 1989; Glancy and Balaban, 2012). The mitochondria-ER Ca^2+^ transfer is proposed to be mediated by the Ca^2+^ channel inositol 1,4,5-trisphosphate receptor Ins(1,4,5)P_3_R on the ER, the voltage-dependent anion-selective channel protein 1 (VDAC1) on the mitochondrial outer membrane, and the mitochondrial Ca^2+^ uniporter (MCU) on the mitochondrial inner membrane (Rowland and Voeltz, 2012). It is thought that the mitochondria-ER contact sites provide a high local concentration of Ca^2+^ for mitochondrial membrane proteins that require Ca^2+^ binding for their functions but cannot bind enough Ca^2+^ at cytoplasmic concentrations. Recently studies begin to suggest that Miro may play an important role in regulating intramitochondrial Ca^2+^ levels. Miro overexpression leads to increased Ca^2+^ uptake by mitochondria upon ER store depletion (Saotome et al., 2008), whereas a mutant form of Miro defective in Ca^2+^ binding decreased the influx of Ca^2+^ into mitochondria (Chang et al., 2011). The mechanism by which Miro regulates intramitochondrial Ca^2+^ level is not understood. It would be interesting to test possible interaction between Miro and the molecules described above that mediate ER to mitochondria Ca^2+^ transfer. In mammalian cells, Mfn2 has been shown to be a component of the mitochondria-ER contact sites (de Brito and Scorrano, 2008). Given the known Miro-Mfn interaction, it is possible that through Mfn2 interaction Miro might regulate mitochondria-ER communication. Since local Ca^2+^ flux can cause Ca^2+^ overload and stimulate apoptosis by opening the mitochondrial permeability transition pore (MPTP), leading to cytochrome *c* release, propagation of the caspase cascade and ultimately apoptosis, the function of Miro in regulating intramitochondrial Ca^2+^ homeostasis may be highly relevant to the pro-apoptotic effects of Miro discussed later.

## Regulation of microtubule dynamics, cell cycle activation, and CNS repair by Miro

In the original study of Miro function in *Drosophila*, it was found that *dmiro* mutants exhibited altered structures of synaptic boutons at the NMJ (Guo et al., 2005). To determine whether the altered bouton structure correlates with an abnormal MT cytoskeleton, the authors examined presynaptic MTs. Compared to control NMJs which showed robust presynaptic MT bundles extending through the entire NMJ and easily identifiable MT loops within large synaptic boutons, *dmiro* mutants exhibited reduced number of MT loops and their presynaptic MT bundles often failed to extend into the last synaptic bouton of axonal branches, suggesting that dMiro mutations altered presynaptic MT cytoskeleton organization, which may contribute to the abnormal synaptic bouton structure. Further studies indicated that *dmiro* mutations selectively affect the MT, but not the actin cytoskeleton. In a recent study of mechanisms of CNS repair using traumatic injury to the ventral midline of the embryonic *Drosophila* CNS as a model, it was found that *dMiro* was significantly downregulated at the mRNA level during CNS repair (Bossing et al., 2012). Further experimentation showed that ectopic Miro expression prevented midline divisions after damage, whereas Miro depletion destabilized cortical β-tubulin and increased cell divisions. Disruption of cortical MTs, either by chemical depolymerization or by overexpression of monomeric tubulin, also triggers ectopic mitosis in the CNS midline. These results indicate that upon injury, the integrity of the MT cytoskeleton regulated by the MT-stabilizing Miro plays an instrumental role in controlling cell division in the CNS midline neural precursor cells. This study further suggests that the function of Miro may not be restricted to postmitotic neurons in the CNS. Intriguingly, mutational effects of Miro on embryogenesis in *Arabidopsis thaliana* has suggest that Miro cam serve an early developmental role (Yamaoka and Leaver, 2008).

The molecular mechanism by which Miro regulates the stability of the MT cytoskeleton is completely unknown. Given that much of Miro function, from mitochondrial trafficking to mitochondrial morphogenesis, depends on the MT cytoskeleton, it is possible that Miro may modulate or interact with proteins that directly regulate MT dynamics (Figure 1). Candidate mediators include MT-binding proteins such as tau, and enzymes that regulate the phosphorylation, acetylation, or glycosylation of tau. Consistent with this notion, it was recently shown that inhibition of Miro alters the phosphorylation status of tau in *Drosophila* photoreceptor neurons through activation of tau kinase PAR-1 (Iijima-Ando et al., 2012).

## Role of Miro alterations in neurological disorders

A disproportionately large percentage of our body energy is used to fuel brain function. Unlike other cells, neurons have limited ability to generate ATP through glycolysis and hence rely heavily on mitochondria-based energy production (Bolanñs et al., 2010; Van Laar et al., 2011). In addition to bioenergetics, mitochondria play other physiological roles essential for neurons, such as buffering of Ca^2+^ influxes elicited by neurotransmission (Werth and Thayer, 1994; Tang and Zucker, 1997). These effects make mitochondria particularly important for brain function and may explain the prominent manifestation of neurological impairments in mitochondrial diseases (Wallace, 2005; Beal, 2007; Mattson et al., 2008). Given the importance of Miro in regulating the transport, morphology, Ca^2+^ homeostasis, and other aspects of mitochondrial physiology, it is not surprising that alteration of Miro function has been associated with disease conditions such as PD (Wang et al., 2011; Liu et al., 2012), amyotrophic lateral sclerosis (ALS; Mórotz et al., 2012), and schizophrenia (Ogawa et al., 2014).

In the first functional characterization of human Miro proteins, it was found that overexpression of the constitutively active form of Miro1 led to increased rate of apoptosis in cultured non-neuronal cells (Fransson et al., 2003). In *Drosophila*, overexpression of dMiro in the brain was found to be toxic, and caused an age-dependent loss of dopaminergic neurons, the cell types that are selectively affected in PD (Liu et al., 2012). It remains to be determined the relative contribution of altered mitochondrial transport, morphology, Ca^2+^ homeostasis, or MT stability to this apoptotic effect caused by elevated Miro activity. In a *Drosophila* model of PD associated with loss of function of the mitochondria-localized Pten-induced kinase 1 (PINK1), reduction of function of Miro as well as components of the transport machinery effectively rescued the degenerative phenotype in dopaminergic neurons seen in *PINK1* mutant, suggesting that at least in this setting altered mitochondrial transport contributes to PINK1 pathogenesis (Liu et al., 2012). Indeed, loss or gain of function of PINK1 had profound effects on the transport of axonal mitochondria in *Drosophila* larval motor neurons or mammalian hippocampal neurons. Biochemically, it was found that PINK1 and another PD associated gene product, Parkin, acted together to promote the degradation of Miro *in vivo* in *Drosophila* tissues, or in cultured mammalian cells treated with the mitochondrial toxin CCCP (Wang et al., 2011; Liu et al., 2012). The exact biochemical mechanism by which the PINK1/Parkin pathway regulates Miro stability remains to be established, as there is no consensus on whether Miro is a direct substrate of PINK1-mediated phosphorylation or whether PINK1-mediated phosphorylation of Miro is a prerequisite for the regulation of Miro stability by the PINK1-Parkin pathway (Wang et al., 2011; Liu et al., 2012; Birsa et al., 2014).

The PINK1/Parkin pathway has been implicated in mitochondrial quality control, during which PINK1 becomes stabilized on damaged mitochondria, recruits Parkin from the cytosol, and targets damaged mitochondria for clearance by autophagy (mitophagy; Narendra and Youle, 2011). In HeLa cells, the loss of hMiro promoted perinuclear clustering of mitochondria and facilitated autophagy of damaged mitochondria, effects previously associated with activation of the PINK1/Parkin pathway (Liu et al., 2012). Interestingly, a recent study found that hMiro1 could stabilize phospho-mutant versions of Parkin on the mitochondrial outer membrane, suggesting that hMiro may be part of a Parkin receptor complex (Birsa et al., 2014). The implication of Miro in the mitochondrial quality control process directed by the PINK1/Parkin pathway and in PD pathogenesis opened up new directions for understanding the regulation and function of Miro in the nervous system, and raised the possibility that dysfunction of Miro may be broadly involved in the pathogenesis of other neurological disorders, where aberrant mitochondrial distribution, morphology, and function have been observed early in the disease process. Supporting the latter notion, a recent study in *Drosophila* showed that inhibition of Miro activated the PAR-1/MARK family kinases and promoted the pathological phosphorylation of tau (Iijima-Ando et al., 2012). Aberrant phosphorylation of tau has been broadly implicated in neurodegenerative diseases known as tauopathies, including AD, frontotemporal dementia, progressive suprenuclear palsy, etc., and activation of the PAR-1/MARK-tau pathway has been observed in AD subjects and animal models (Chin et al., 2000; Wang et al., 2007; Iijima-Ando et al., 2009; Zempel et al., 2010; Yu et al., 2012). Supporting the involvement of Miro alteration in the pathogenesis of other neurological diseases, DISC1 (Disrupted in Schizophrenia 1), a candidate risk factor for schizophrenia, bipolar disorder, and depression, has been shown to interact with TRAK1 and Miro1 to promote axonal mitochondrial transport, and a putative disease-associated variant 37W impairs DISC1 function in this process (Ogawa et al., 2014). Furthermore, the P56S variant of vesicle-associated membrane protein associated protein B (VAPB), which is associated with dominantly inherited ALS type 8, has been shown to decrease the association between tubulin and Miro by increasing cytosolic Ca^2+^ level, thereby disrupting anterograde mitochondrial transport in neuronal axons, and a Ca^2+^-insensitive form of Miro1 rescued the effects of VAPB-P56S on axonal mitochondria transport (Mórotz et al., 2012). Future studies linking the intracellular signaling pathways implicated in the pathogenesis of various neurological diseases with the biochemical or biophysical alterations in Miro proteins will undoubtedly offer novel insights into the regulation and function of Miro in the nervous system, the contribution of Miro dysfunction to disease pathogenesis, and the development of Miro-based therapeutic avenues.

## Conclusion

Mitochondria play essential roles in normal neuronal physiology, from energy production to Ca^2+^ buffering to synaptic differentiation and plasticity. The intracellular distribution of mitochondria needs to be precisely matched to the demand for these organelles, a task particularly difficult for neurons due to their highly polarized morphology and their dynamic patterns of neuronal activity and synaptic plasticity *in vivo*. Because abnormal mitochondrial distribution and function has been consistently observed at early stages of neurodegenerative disorders such as AD and PD as well as neuropsychiatric disorders, understanding the genetic control of mitochondrial distribution in neurons has assumed a high priority in neuroscience research. The identification of the Miro/Milton/Khc complex as a conserved mitochondrial transport machinery has offered tremendous insights into the role of the evolutionarily conserved Miro proteins in mitochondrial trafficking in neurons. Studies of Miro proteins in a number of organisms have suggested that the function of Miro may go beyond transport of mitochondria in neurons. Newly revealed functions of Miro proteins include mitochondrial morphogenesis, mitochondria-ER communication, MT dynamics, and apoptosis. Futures studies of Miro function in diverse experimental systems, from model organisms such as yeast and *Drosophila* to patient-derived neurons should further our understanding of the myriad function of Miro in the nervous system and its relevance to human health and disease.

## Conflict of interest statement

The authors declare that the research was conducted in the absence of any commercial or financial relationships that could be construed as a potential conflict of interest.
